# Topography of Striate-Extrastriate Connections in Neonatally Enucleated Rats

**DOI:** 10.1155/2013/592426

**Published:** 2013-10-03

**Authors:** Robyn J. Laing, Jurate Lasiene, Jaime F. Olavarria

**Affiliations:** ^1^Department of Psychology, and Behavior and Neuroscience Program, University of Washington, P.O. Box 351525, Seattle, WA 98195-1525, USA; ^2^Laboratory for Motor Neuron Disease, RIKEN Brain Science Institute, 2-1 Hirosawa, Wako-Shi, Saitama Prefecture 351-0198, Japan

## Abstract

It is known that retinal input is necessary for the normal development of striate cortex and its corticocortical connections, but there is little information on the role that retinal input plays in the development of retinotopically organized connections between V1 and surrounding visual areas. In nearly all lateral extrastriate areas, the anatomical and physiological representation of the nasotemporal axis of the visual field mirrors the representation of this axis in V1. To determine whether the mediolateral topography of striate-extrastriate projections is preserved in neonatally enucleated rats, we analyzed the patterns of projections resulting from tracer injections placed at different sites along the mediolateral axis of V1. We found that the correlation between the distance from injection sites to the lateral border of V1 and the distance of the labeling patterns in area 18a was strong in controls and much weaker in enucleates. Data from pairs of injections in the same animal revealed that the separation of area 18a projection fields for a given separation of injection sites was more variable in enucleated than in control rats. Our analysis of single and double tracer injections suggests that neonatal bilateral enucleation weakens, but not completely abolishes, the mediolateral topography in area 18a.

## 1. Introduction

Visual information is processed along ascending neuronal pathways that maintain the point-to-point topography of the retinofugal projections. Much work has focused on understanding how topography is established in the projections from ganglion cells to the superior colliculus and thalamus [1–3], but less is known about how higher-order pathways develop. In particular, there is little information about the mechanisms guiding the development of retinotopically organized connections, known as topographic maps, between primary visual cortex (V1, striate cortex, area 17) and higher extrastriate visual areas. Recent studies support the idea that development of cortical areas and their organized interconnections may depend on a combination of activity-independent cortical factors, such as genetically determined guidance labels, and activity-dependent mechanisms driven by sensory input [4–8]. 

During development, interhemispheric callosal connections in V1 are established between opposite cortical loci that are retinotopically matched (i.e., they represent the same visual coordinates [9–11]). Olavarria and colleagues proposed that the temporal retina, through a system of bilateral projections, promotes the stabilization of callosal linkages that, while retinotopically corresponding, are arranged in a nonmirror symmetric pattern with respect to the brain midline [9, 10, 12]. However, in the absence of retinal input callosal connections are established between opposite cortical loci that are mirror-symmetric with respect to the midline [12, 13]. This reversal in the callosal map induced by enucleation provides evidence that retinal input can specify cortical topography. However, it remains unclear whether or not retinal input plays a major role in the establishment of topographically organized *intra*hemispheric connections between V1 and surrounding extrastriate visual areas. Data supporting a role for retinal input come from a recent study in mice showing that both neonatal enucleation and anophthalmia induce topographic anomalies in striate-extrastriate projections [14]. 

Numerous anatomical and physiological studies in normal rats have shown that V1 is surrounded by at least 10 extrastriate visual areas [15, 16], which have been named according to their location relative to V1 (see Figure 2(a)). Each of these areas contains a representation of the opposite visual hemifield and receives direct, retinotopically organized, projections from V1 (see [17, 18], for reviews). Moreover, callosal connections form a dense band that straddles the V1/18a border and a series of ring-like callosal bands that separate adjoining extrastriate visual areas in area 18a [19–23] (Figure 2(a)). These callosal rings provide fixed reference landmarks for locating and identifying the various visual areas in area 18a of normal rats. 

The pattern of striate-extrastriate projections in normal rats is remarkably consistent from animal to animal [17, 19]. Moreover, tracer injections placed into different loci in V1 produce essentially the same extrastriate projection patterns, except that the projection fields translocate locally according to the retinotopic map in each extrastriate visual area. This has made it possible to obtain information about the topography of striate-extrastriate connections in normal rats using single tracer injections into V1 [19]. In contrast to normal rats, we recently reported that neonatal enucleation induces highly irregular and variable patterns of striate-extrastriate and callosal projections [23]. However, this previous study, based on the analysis of projection patterns produced by single, restricted injections of anatomical tracers into V1, did not report the effects of enucleation on the topography of these projections. In the present study, we have addressed this issue by analyzing data from single tracer injections into V1, as well as connection patterns resulting from pairs of discrete tracer injections placed at different locations in V1 of the same animal.

## 2. Materials and Methods

Our study is based on data obtained from a total of 24 Long-Evans pigmented rats. Pregnant animals were monitored several times daily, and the births of the litters were determined to within 12 hours. Sixteen rat pups were anesthetized with isoflurane (2–4% in air) and binocularly enucleated within 24 hours of birth (BE0). After recovering from the anesthesia, pups were returned to their dams. In addition, 8 rats were used for analyzing striate-extrastriate projections in normal adult animals. All surgical procedures were performed according to protocols approved by the Institutional Animal Care and Use Committee (IACUC) at the University of Washington.

### 2.1. Tracer Injections

In both control and enucleated animals, striate-extrastriate projections were revealed following restricted tracer injections into striate cortex, while the distribution of callosal connections was demonstrated in the same hemisphere following multiple injections of a different tracer in the opposite hemisphere. Anatomical experiments in all normally reared and enucleated rats took place when the animals were at least 1 month old. Tracer injections were made under isoflurane anesthesia (2–4% in air). To study the topography of striate-extrastriate connections, animals received restricted injections of various tracers into different places of striate cortex, including biotinylated dextran amine (BDA, 10% in DW, Molecular Probes, Eugene, OR, USA), which is predominantly transported anterogradely, horseradish peroxidase (HRP, Sigma Co, 25% in saline), which is transported both anterogradely and retrogradely, and fluorescent tracers (rhodamine beads, RB, or green beads, GB, LumaFluor, Naples, FL, USA concentrated stock solution), which are transported retrogradely. Small volumes (0.05–0.1 *μ*L) of these tracers were injected into striate cortex of the right hemisphere (approx. 2.9–4.2 mm from the midline; 0.1–2.0 mm anterior to the lambda suture). In all cases analyzed the tracer injections were restricted to grey matter. The size of single injections of BDA and HRP was estimated as described previously [23]. High levels of fluorescence from GB and RB injections were typically restricted to the injection sites because diffusion of these tracers is low. To reveal the overall pattern of callosal connections, multiple injections (12–15 injections; total volume approx. 4.0 *μ*L) of either HRP or bisBenzimide (BB, Sigma Co., 10% in DW) were placed over visual cortex of the left hemisphere [24]. All tracers were pressure-injected through glass micropipettes (50–100 *μ*m tip diameter). Data from some cases injected with BDA were presented in Laing et al. [23].

### 2.2. Histochemical Processing

After a survival period of 2 days, the animals were deeply anesthetized with pentobarbital sodium (100 mg/kg i.p.) and perfused through the heart with 0.9% saline followed by 4% paraformaldehyde in 0.1 M phosphate buffer (PB, pH 7.4). After the brain was removed from the skull, the cortical mantle to be analyzed was separated from the brainstem, flattened between glass slides, and sectioned tangentially (60 *μ*m thick sections) as described previously [23]. The flattening procedure was done with great care to ensure that both striate and extrastriate cortices were contained in the tangential sections. The thalamus was cut into 60 *μ*m thick coronal sections. If fluorescence was combined with either BDA or HRP, patterns were analyzed in alternate series of sections. Sections in the series examined only for fluorescence were mounted on slides and analyzed under epifluorescence without further processing. However, sections in the series processed for BDA or HRP were also often analyzed for epifluorescence because the fluorescent labeling in these sections was similar to that in sections not processed for these tracers. This allowed a direct correlation of the spatial location of both fluorescent and nonfluorescent labeling in the same section. BDA labeling was revealed using the standard avidin-biotin-peroxidase protocol (Vectastain Elite ABC kit, Vector Laboratories, Burlingame, CA, USA) and 0.01% H_2_O_2_ in 0.05% 3,3′-diaminobenzidine, with cobalt or nickel intensification; sections were then mounted, dehydrated, defatted, and coverslipped. HRP labeling was revealed using tetramethylbenzidine as the chromogen [25].

### 2.3. Data Acquisition and Analysis

**Figure 1:**
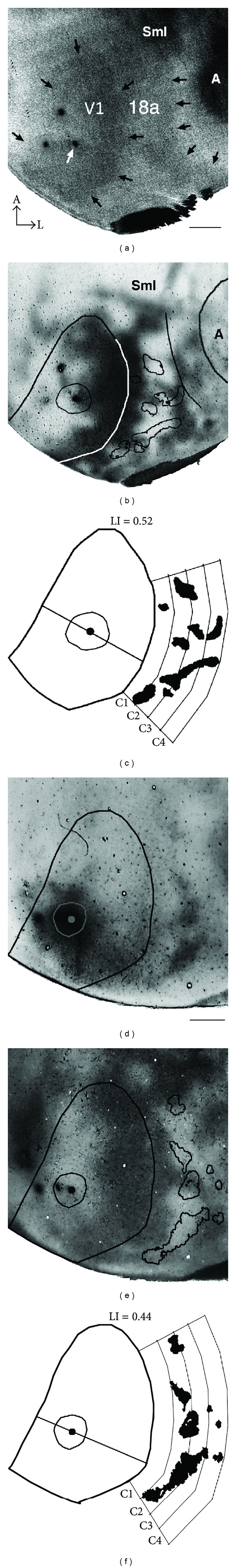
Correlating the patterns of striate-extrastriate projections with the patterns of myelination and visual callosal connections; procedure for calculating LI. (a) Myelin pattern from a rat (BEM-2) enucleated at birth. Black arrows indicate borders of V1 and area 18a. A, auditory cortex; Sml, somatosensory cortex. White arrow indicates BDA injection site. Neighboring dark spots correspond to injections of other tracers. (b) Callosal pattern revealed following multiple injections of HRP in the contralateral hemisphere in the same enucleated rat. Dark areas correspond to dense accumulations of retrogradely labeled somas and anterogradely labeled axons. The profiles of V1, 18a, and auditory cortex were drawn from the myelin pattern in (a). Regions outlined in black represent BDA-labeled projection fields drawn from a section processed for BDA. Black dot indicates the location of the BDA injection, and the circular outline estimates the size of tracer diffusion (see Section 2). (d) Pattern of BDA-labeled projections in another enucleated rat (case M6e4). Grey dot and grey outline indicate the injection and diffusion of BDA, respectively. Border of V1, outlined in black, was determined from the callosal pattern (e) in the same case. (e) Pattern of HRP-labeled callosal connections in the same enucleated rat. Black lines in lateral extrastriate cortex outline the BDA-labeled projections shown in (d). ((c) and (f)) Diagrams illustrating the compartments (C1–C4) in area 18a used in LI calculations. The LI for each case is indicated. Line passing through injection site was used to calculate the distance from the injection site to the lateral border of V1. Inset in (a) indicates orientation, A: anterior, and L: lateral. Scale bars = 1.0 mm.

Digital images of the BDA- and HRP-labeling patterns in histological sections were obtained by scanning the sections at 2400 dpi using an Epson 4990 scanner. The distribution of cells labeled with fluorescent tracers was analyzed using a microscope equipped with a motorized stage (LEPCO) controlled by a Dell XPS T500 computer and a graphic system (Neurolucida, MicroBrightField, Williston, VT, USA). The borders of areas 17 and 18a [26–28] were identified in the myelin pattern by scanning unstained tangential sections [23, 29] (Figure 1(a)). In both normal and enucleated rats, area 18a is the target of virtually all projections from V1 to lateral extrastriate cortex [23], so in this report we will consider the terms “lateral extrastriate cortex” and “area 18a” as synonymous. Further information for identifying the location of the border of areas 17 and 18a in control and enucleated rats came from analyzing landmarks provided by the overall callosal pattern in visual cortex and the relation that these landmarks have with the borders of areas 17 and 18a as revealed in the myelination patterns [23, 24, 30] (Figure 1).

**Figure 2:**
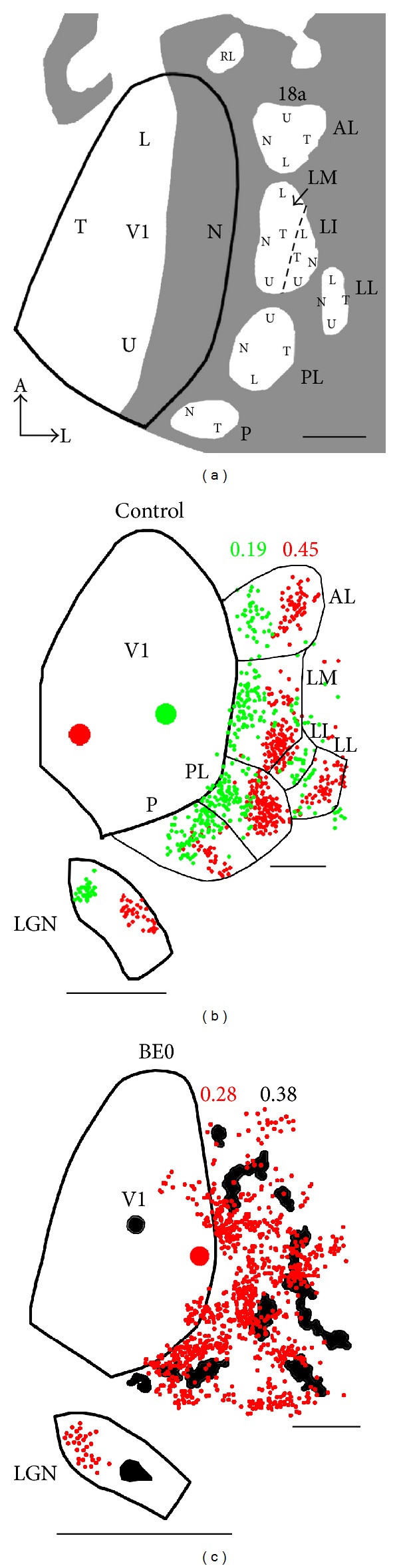
Topography of striate-extrastriate connections in normal and enucleated rats. (a) Diagram of the distribution of callosal connections and visual areas in lateral striate cortex of normal rats. The border of V1 is outlined in black; callosal pattern is indicated in grey, while acallosal areas are indicated in white. The diagram summarizes previous studies (see references in text) of the overall topographic organization of V1 and some visual areas in lateral extrastriate cortex (area 18a). Cortical regions representing upper, lower, nasal and temporal portions of the visual field are indicated by U, L, N, and T, respectively. RL: rostrolateral, AL: anterolateral, LM: lateromedial, LI: laterointermediate, LL: laterolateral, PL: posterolateral, and P: posterior. Modified from [31]. (b) Data from a normal rat (case C21) that received injections of GB (green dot) and RB (red dot) in V1. Cells labeled with either RB or GB are indicated by small red or green dots, respectively. Approximate location of the border of visual areas in area 18a is indicated by thin black lines. (c) Data from an enucleated rat (case M4e9) that received injections of RB (red dot) and BDA (black dot) in V1. BDA-labeled fields are indicated in black. The laterality index for each tracer injection in (b) and (c) is indicated. Insets in (b) and (c) show labeled fields in the dLGN. Callosal patterns, as well as striate projection fields in medial extrastriate cortex are not represented in (b) and (c). Inset in (a) indicates orientation, A: anterior, L: lateral. Scale bars = 1.0 mm.

The locations of the injection sites within striate cortex were confirmed by analyzing the distribution of labeled fields within the ipsilateral dorsal lateral geniculate nucleus of the thalamus (dLGN) [32–36]. The distance between injections of different tracers ranged from 0.8 to 1.6 mm. These distances were judged adequate for studying the topography of striate projections because the injections produced separate labeling fields in the dLGN (Figures 2(b) and 2(c)). Tangential sections throughout the depth of the cortex were analyzed to ensure that injections were restricted to grey matter. 

Using Adobe Photoshop CS2 (Adobe Systems), digitized images of both anatomical tracer and myelin labeling patterns from the same animal were carefully aligned with each other using the border of V1, the edges of the sections, blood vessels, and other fiducial marks. Cells labeled retrogradely by the injections of RB and GB were represented by red and green dots, respectively. Callosal patterns labeled with BB were represented by outlining the areas containing dense accumulations of labeled cells. To illustrate the patterns of callosal connections labeled with HRP or striate projections resulting from restricted injections of HRP or BDA, thresholded versions of these patterns were prepared after first applying a median filter to reduce noise, followed by a high-pass filter to remove gradual changes in staining density across the entire digital image. The same filter parameters and thresholding levels were applied to all control and enucleated animals, and thresholded versions were visually inspected to confirm that they accurately represented the labeling pattern observed in the tissue sections. Figures were prepared using Adobe Photoshop CS2, and all imaging processing used, including contrast enhancement and intensity level adjustments, were applied to the entire images.

#### 2.3.1. Quantitative Analysis

We analyzed the distribution of labeled fields in lateral extrastriate cortex resulting from injections placed at different mediolateral locations in V1. The distance of the injection sites from the lateral border of V1 was expressed as the percentage of the width of V1 measured along a line passing through the injection site and perpendicular to the V1/18a border (Figures 1(c) and 1(f)). To evaluate the overall distribution of the labeling pattern in area 18a with respect to the lateral border of V1, we divided lateral extrastriate cortex into four compartments (C) of equal width and numbered C1 to C4 from medial to lateral. These compartments were drawn parallel to the lateral border of V1 and to each other (Figures 1(c) and 1(f)). For each tracer injection, we calculated a laterality index (LI) using the following formula:

(1) $$\begin{array}{c} \text{LI}=\frac{\left[\left(\text{C}4-\text{C}1\right)+2/3\left(\text{C}3-\text{C}2\right)+N\right]}{2N}, \end{array}$$

where C*n* is the number of pixels within compartment *n* and *N* is the total number of labeled pixels in area 18a. This index ranges from 0.0 to 1.0, values indicating that 100% of the labeling produced by a particular injection in V1 is contained within C1 or C4, respectively. Changes in LIs produced by pairs of tracer injections into V1 of the same animal were evaluated statistically using paired *t*-tests. 

## 3. Results

 The diagram in Figure 2(a), based on numerous anatomical and physiological studies in normal rats (see references above), illustrates the arrangement of visual areas in lateral extrastriate cortex, their internal topography, and their relationship to the overall pattern of callosal connections. This diagram shows that, with the exception of the small area LI, the maps in all lateral extrastriate areas are mirror images of the map in V1 along the mediolateral axis (from temporal to nasal visual field representation). In contrast, the maps of the anteroposterior axis in V1 (from lower to upper visual field representation) differ among the areas; while the maps in areas AL, P, LI, and LL show the same polarity as the map in V1, the polarity is inverted in areas AL and PL.

As it is difficult to identify with certainty specific extrastriate areas in enucleated rats due to marked abnormalities in striate-extrastriate and callosal connections [23, 37], we did not pursue the study of the effect of enucleation along the anteroposterior axis. Instead, we focused our attention on studying whether or not displacements of the injections along the mediolateral axis of V1 in enucleated rats lead to mirror-image displacements of the overall labeling patterns in lateral extrastriate cortex. To this end we correlated the distances from the injection sites to the V1/18a border with the LI calculated for each labeling pattern in area 18a of control and enucleated rats (see Section 2).

**Figure 3:**
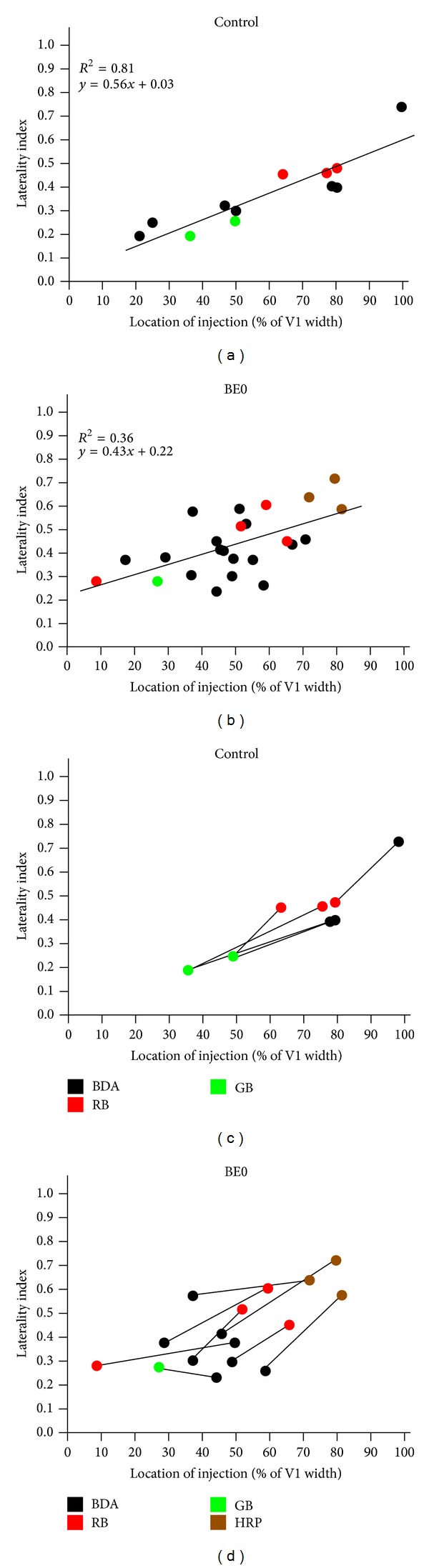
Laterality indices for normal and enucleated rats. ((a) and (b)) Scatterplots correlating the LI values calculated for single injections with the distance of the injection sites to the lateral border of V1, expressed as a percentage of the width of V1 (see Figures 1(c) and 1(f)). Black, red, green, and brown dots represent injections of BDA, RB, GB, and HRP, respectively. ((c) and (d)) Comparing the separation of labeling patterns in area 18a produced by injections of different tracers into different regions of V1 in the same animal. Black lines connect data from the pair of injections in the same animal.

As expected, in control rats we observed a high correlation (*R* ^2^ = 0.81) between the distance of injection sites from the lateral border of V1 and the LIs of the resulting labeling patterns in area 18a (Figure 3(a), 14 injection sites from 8 rats). This figure shows that, as the distance of the injections from the lateral border of V1 increased, the overall labeling patterns moved more laterally in lateral extrastriate cortex, resulting in higher LIs. A positive, although weaker, correlation (*R* ^2^ = 0.36) was observed in enucleated rats (Figure 3(b), 24 injections from 16 rats). These results are consistent with the high variability of striate-extrastriate projections in enucleated rats [23] and suggest that, in spite of this variability, the overall labeling patterns in area 18a of enucleated rats tend to move laterally when the injection sites are displaced medially in V1.

Due to the high animal-to-animal variability of striate-extrastriate projection patterns in BE0 rats, it is typically not possible to transform the pattern in one animal into that of another simply by displacing the fields in a consistent manner, as is often possible in normal animals [23]. This variability may to some extent obscure the mediolateral topography in area 18a of BE0 rats when the data from different animals are pooled together, as in Figure 3(b). To examine this possibility, we compared the changes in LIs resulting from pairs of injections placed at different locations in V1 of the same animals. 

Figure 2(b) presents the results from a control rat that received restricted injections of GB and RB at different distances from the lateral border of V1. The arrangement of the injection sites (red dot = RB, green dot = GB) permits analyzing the mapping along the mediolateral axis in V1. In lateral extrastriate cortex, red and green dots represent individual cells retrogradely labeled with either RB or GB, respectively. Figure 2(b) shows that when the injection site is displaced from lateral (green dot) to medial (red dot) in V1, the labeled fields in extrastriate cortex move in a mirror-image fashion with respect to the border of V1, as predicted from the mapping summarized in Figure 2(a). Thus, the cells labeled by the GB injections accumulate in medial portions of lateral extrastriate cortex, near the lateral border of V1, while the cells labeled by the RB injection accumulate more laterally in extrastriate cortex. Consistent with these observations, the LI associated with the GB injection (0.19) is smaller than that associated with the RB injection (0.45). 

Results from a double-injection experiment in a BE0 rat are shown in Figure 2(c). This animal received an injection of RB close to the lateral V1 border and an injection of BDA further medially. Both injections produced labeled fields distributed over broad regions in lateral extrastriate cortex, and the overall arrangement of labeled fields was the same for both tracers. The LI calculated for the distribution of RB-labeled cells (0.28) is smaller than that for the BDA-labeled pattern (0.38), suggesting that the mediolateral displacement of the overall pattern of labeling in area 18a mirrors the displacement of the injection sites in V1 with respect to the lateral border of V1. 

Analysis of a pair of injections in control rats (Figure 3(c)) reveals a significant increase of the LIs (*P* < 0.01) calculated for the medial injections (*M* = 0.49, SD = 0.14) compared to the LIs calculated for the lateral injections (*M* = 0.27, SD = 0.12) (*n* = 5, paired *t*-test). In BE0 rats (Figure 3(d)), the LIs calculated for the medial injections (*M* = 0.50, SD = 0.15) were also significantly larger (*P* < 0.01) than the LI calculated for the lateral injections (*M* = 0.32, SD = 0.10) (*n* = 8, paired *t*-test). However, comparison of the slopes of the lines connecting each pair in the data sets shows that the slopes tend to be more variable in BE0 rats than in control (slopes range from 0.49 to 1.39 in control rats and from −0.23 to 1.41 in BE0 rats). These data suggest that, in spite of abnormalities in the projection patterns [23], the basic mirror-image topography of area 18a is largely preserved in BE0 rats. They also suggest that for a given separation of the injection site in V1, there is more variability in the separation of extrastriate labeled fields in BE0 than in control rats. 

 In the dLGN, we observed that restricted injections in striate cortex produced separate labeled fields in the dLGN (insets in Figures 2(b) and 2(c)), confirming that the areas of effective tracer uptake associated with the cortical tracer injections did not overlap significantly. Compared with normal rats, the labeled fields in the dLGN of enucleated rats appeared larger relative to the size of the dLGN, possibly because neonatal enucleation reduces the size of both striate cortex [23] and dLGN [36, 38]. Our observations in the dLGN are consistent with previous studies indicating that the geniculocortical pathway in enucleated rats and other animals maintains the gross topography observed in normal animals [33–36].

## 4. Discussion

A recent study in adult rats neonatally enucleated at birth showed that single tracer injection into different regions of V1 produce, anomalous and highly variable patterns of striate-extrastriate and callosal projections [23]. This previous study noted that it was typically not possible to transform the pattern in one animal into that of another simply by displacing the fields in a consistent manner. These results are in agreement with a recent study of the effects of enucleation and anophthalmia on the distribution of striate-extrastriate projections in mice [14]. In contrast, in normal animals the arrangement as well as the retinotopic organization of extrastriate areas is highly consistent from animal to animal [17, 19]. Although the distributions of callosal and striate-extrastriate projections in enucleated rats have an overall resemblance to those in normal rats [23], the anomalies and variability in these projections impede the identification of specific visual areas in extrastriate cortex with certainty. It is, therefore, difficult to analyze the effect of enucleation on the topography of individual visual areas. Here, we took advantage of the fact that the representation of the nasotemporal axis of the visual field in nearly all lateral extrastriate visual areas mirrors the representation of this axis in V1 with respect to the lateral V1 border. We were interested in determining whether the basic mediolateral topography in the striate-extrastriate projections was preserved in spite of abnormalities in the projection patterns. Rather than analyzing how injections into different mediolateral locations in V1 change the projections to specific extrastriate regions, we analyzed the displacements of the overall patterns of labeling produced in area 18a by changes in injection sites. We assessed these changes using a laterality index LI, which is based on the proportion of the total labeling pattern that is contained in 4 equally spaced compartments oriented parallel to the lateral border of V1. The value of the LI increased or decreased as the labeling tended to accumulate in lateral or medial regions of area 18a, respectively. 

In normal rats we found a strong correlation between the mediolateral location of the injections and the corresponding LIs, indicating that the sensitivity of this index is adequate for detecting displacements of the labeling patterns produced by even relatively small displacements of the injection sites. A positive correlation was also observed in enucleated rats, but it was weaker than in normal rats. Since this result was based on the analysis of single injections from different animals, the weaker correlation may simply reflect the variability of projection patterns across rats. To examine this possibility, we compared the separation of labeling patterns produced by injections of different tracers into different regions of V1 in the same animal. We found significant, mirror-image separations of area 18a labeling patterns in both control and enucleated rats. However, our data suggest that the separation of labeling patterns in area 18a for a given separation of the V1 injections was somewhat more variable in enucleated rats than in control rats.

Thus, in enucleated rats we were able to detect mirror-image changes in the distribution of overall labeling patterns in area 18a in response to changes in the mediolateral location of tracer injections into V1. However, these changes tended to be more variable in enucleated than in control rats. Together, our analyses of single and double tracer injections suggest that neonatal bilateral enucleation weakens, but not completely abolishes, the mediolateral topography in area 18a.

It is possible that the difference we found between normal and enucleated rats reflects differences in the injections placed in the two groups of animals. This is unlikely because the injections were small compared to the size of striate cortex, arranged similarly along the mediolateral axis in both groups, and similar results were observed with different combination of tracers. Moreover, the separations between the injection sites were judged to be adequate because they produced separate labeled fields in the dLGN.

Previous studies have shown that neonatal bilateral enucleation reverses the topography of visual callosal connections. In normal rats callosal connections interlink opposite cortical loci that are retinotopically matched but located asymmetrically with respect to the brain midline. In contrast, in neonatally enucleated rats callosal links are established between opposite cortical loci that are mirror symmetric with respect to the midline [12, 13]. Moreover, the callosal topography in enucleated rats is less precise than in control rats. These results have led to the proposal that retinal input guides the retinotopically precise ingrowth of callosal axons in visual cortex, whereas the cues that determine the mirror-symmetric callosal maps in enucleates exert only a weak control on the topography of callosal fiber ingrowth [12, 39]. Thus, under normal conditions, these weaker cues would be superseded by stronger influences of retinal origin, leading to a nonsymmetric, retinotopically matched callosal map. 

Our present results suggest that hierarchical, topographic cues may also regulate the organization of striate-extrastriate connections, but in this case the maps that develop in both normal and enucleated rats show similar mediolateral topography rather than opposite topography as is the case with callosal maps. Although our data suggest that mediolateral topography develops in area 18a under either retinally driven cues or cues of central origin, it is important to note that retinal cues appear to be critical for the development of connections patterns that are highly consistent from animal to animal. Thus, retinal input may not only specify the normal internal topography of extrastriate visual areas but may also have an important role in the parcellation of extrastriate cortex into an array of visual areas that is remarkably constant in normal animals. 

How does retinal input influence the development of striate-extrastriate maps? Projections from the dLGN are primarily confined to striate cortex [40–42], so retinal input from the dLGN could reach area 18a via the projections from V1. However, although enucleation reduces the size of both the dLGN and V1 [23, 36, 38, 43], it does not change the basic topography of the dLGN projection to V1 [33, 34, 36]. Retinal input can also reach area 18a via the lateroposterior nucleus of the thalamus (LP) [42, 44], which receives direct projection from the superior colliculus [45]. Thus, enucleation could interfere with the layout of topographic cues in area 18a by disrupting this alternative pathway. Indeed, Négyessy et al. [46] reported that cortical projections from LP were abnormal in neonatally enucleated rats. During normal development, retinal input may regulate the arrangement and topography of extrastriate visual areas by specifying the normal distribution of cortical guidance labels through either activity-dependent or activity-independent cues. For instance, interactions between gradients of EphA/ephrin-A could guide the formation of topographically organized projections from V1 to each extrastriate visual area in a manner similar as they guide the development of the thalamocortical pathway [8]. Central cues operating in the absence of retinal input could specify reduced or distorted gradients leading to the development of anomalous and variable topographic projections between V1 and area 18a. Detecting these gradients and possible changes induced by neonatal enucleation or other manipulations will be specially challenging given the small size of the extrastriate visual areas. Finally, it should be noted that whatever are the mechanisms by which retinal input guides cortical topography, they must exert their effect by postnatal day 6 (P6) because enucleation at P6 or later no longer prevents the development of normal patterns of striate-extrastriate connections [23].
